# Supplementary material for: Proteomic and Metabolomic Analysis of the Quercus ilex–Phytophthora cinnamomi Pathosystem Reveals a Population-Specific Response, Independent of Co-Occurrence of Drought
Source: Biomolecules. 2024 Jan 29;14(2):160. doi: 10.3390/biom14020160 (PMC10887186; doi:10.3390/biom14020160)
Supplement: Supplementary file 1 [file biomolecules-14-00160-s001.zip › Supplementary figures_revised.pptx]

## Slide 1
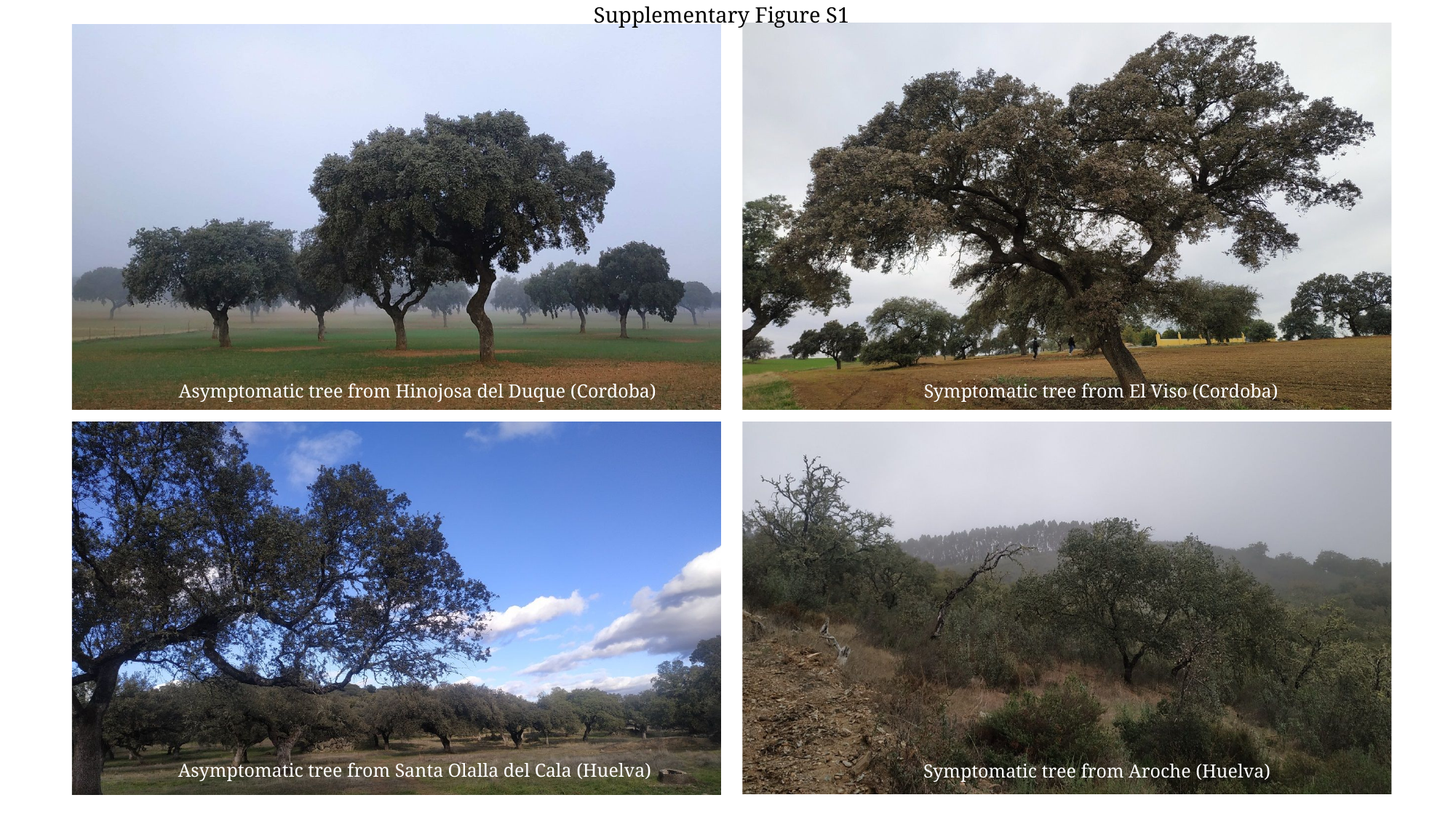

Supplementary Figure S1
Asymptomatic tree from Hinojosa del Duque (Cordoba)
Symptomatic tree from El Viso (Cordoba)
Asymptomatic tree from Santa Olalla del Cala (Huelva)
Symptomatic tree from Aroche (Huelva)

## Slide 2
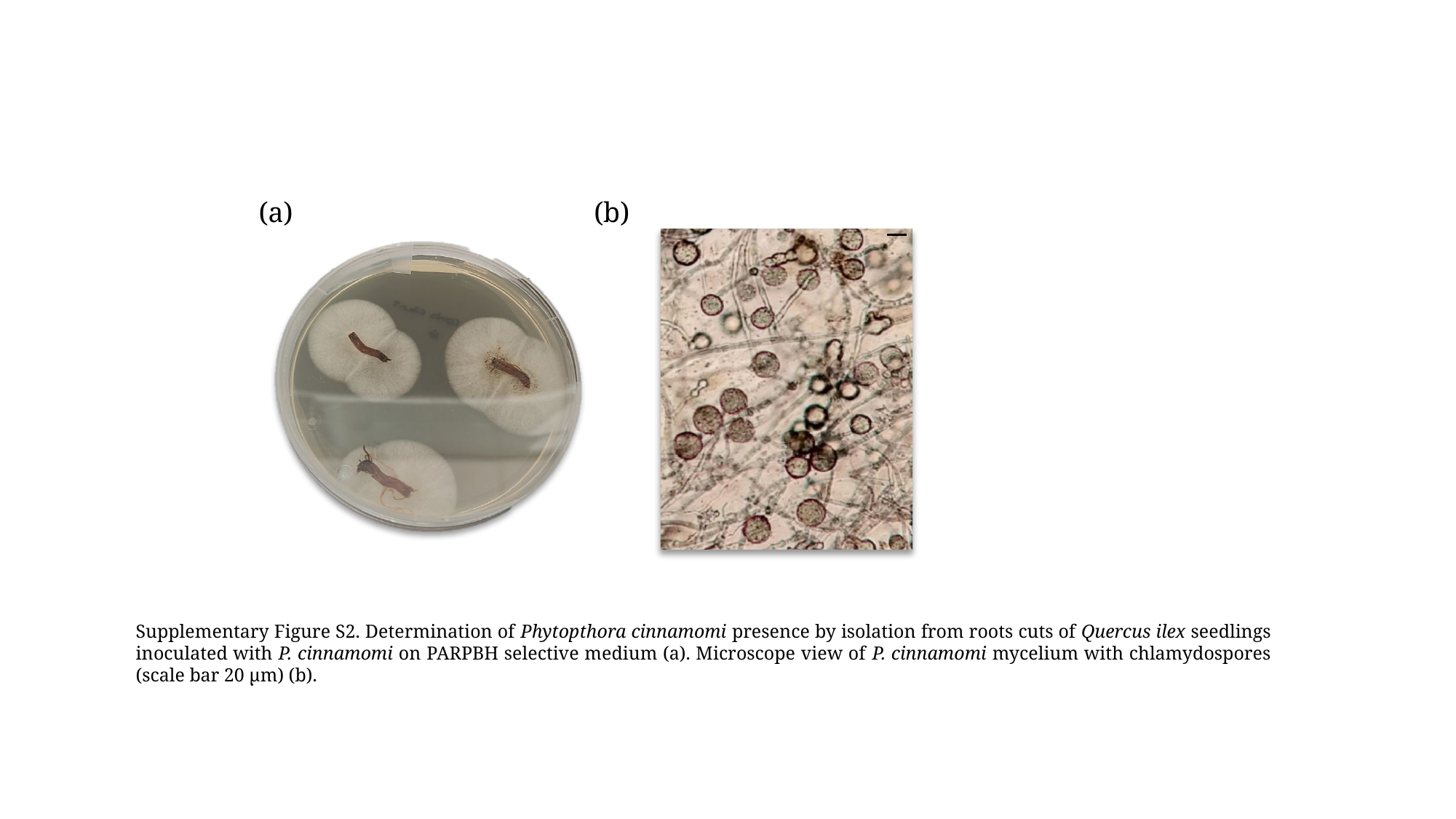

(a)
(b)
Supplementary Figure S2. Determination of Phytopthora cinnamomi presence by isolation from roots cuts of Quercus ilex seedlings inoculated with P. cinnamomi on PARPBH selective medium (a). Microscope view of P. cinnamomi mycelium with chlamydospores (scale bar 20 µm) (b).

## Slide 3
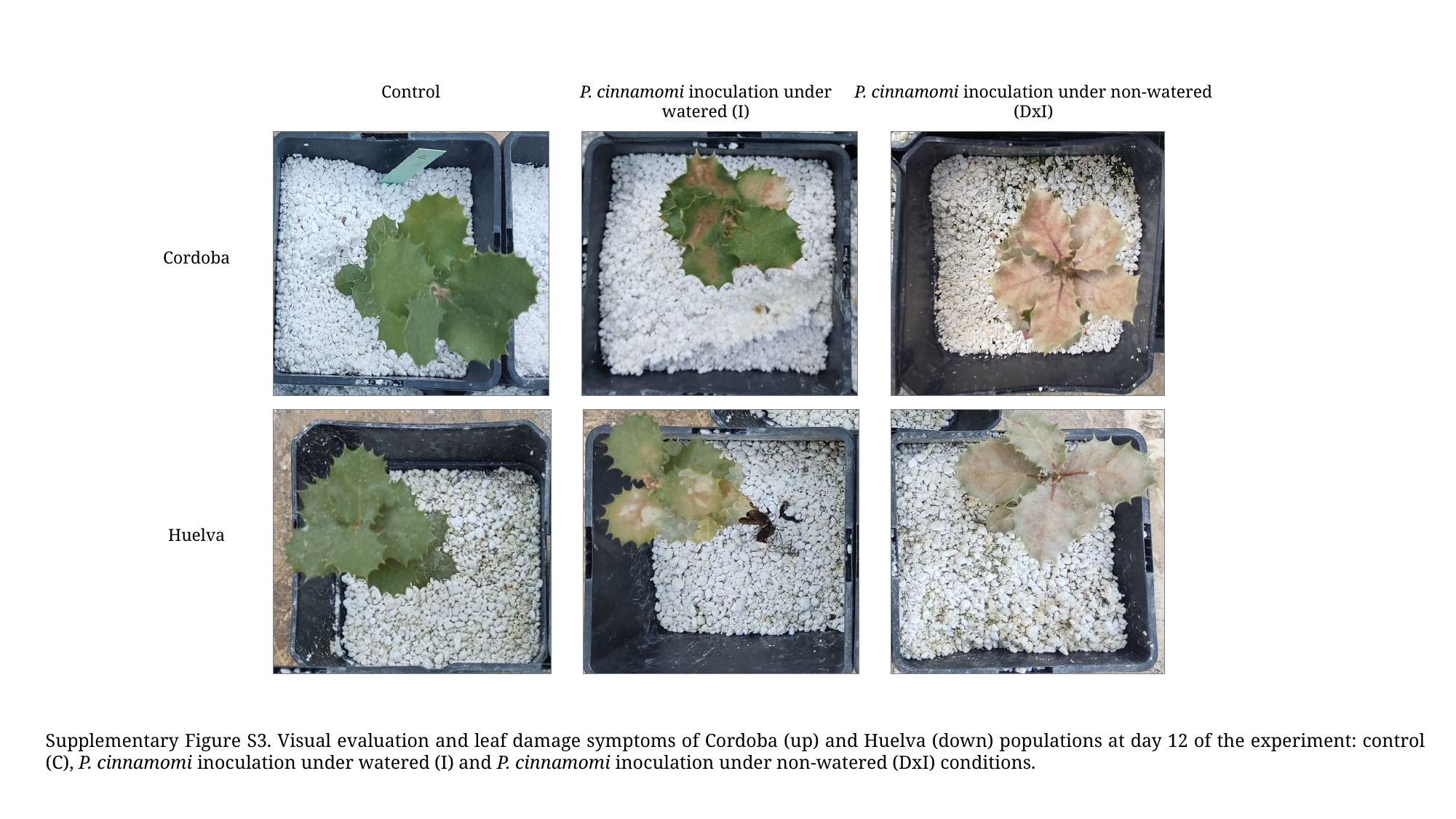

Control
P. cinnamomi inoculation under watered (I)
P. cinnamomi inoculation under non-watered (DxI)
Cordoba
Huelva
Supplementary Figure S3. Visual evaluation and leaf damage symptoms of Cordoba (up) and Huelva (down) populations at day 12 of the experiment: control (C), P. cinnamomi inoculation under watered (I) and P. cinnamomi inoculation under non-watered (DxI) conditions.

## Slide 4
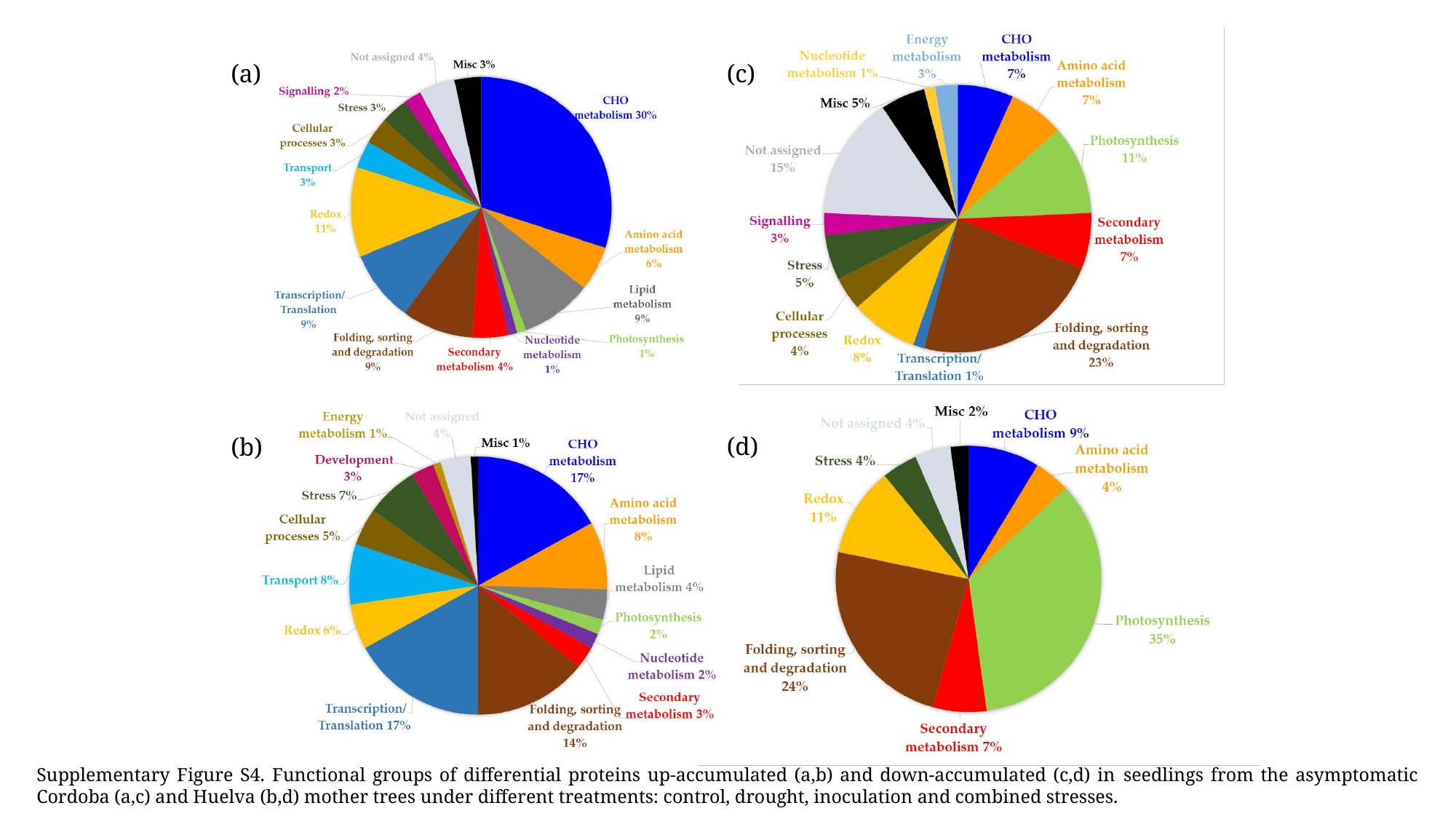

(c)
(a)
(d)
(b)
Supplementary Figure S4. Functional groups of differential proteins up-accumulated (a,b) and down-accumulated (c,d) in seedlings from the asymptomatic Cordoba (a,c) and Huelva (b,d) mother trees under different treatments: control, drought, inoculation and combined stresses.

## Slide 5
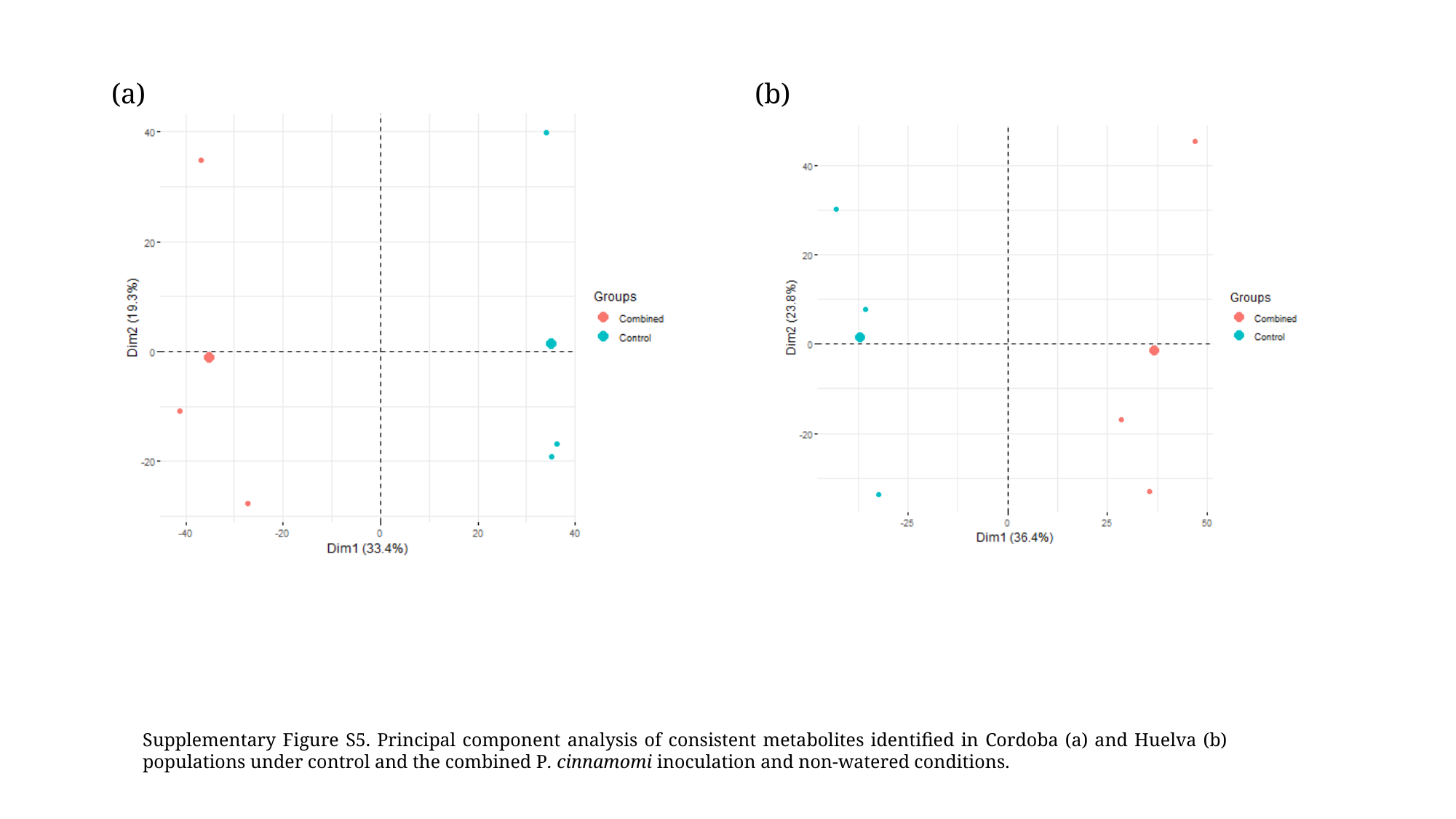

(a)
(b)
Supplementary Figure S5. Principal component analysis of consistent metabolites identified in Cordoba (a) and Huelva (b) populations under control and the combined P. cinnamomi inoculation and non-watered conditions.

## Slide 6
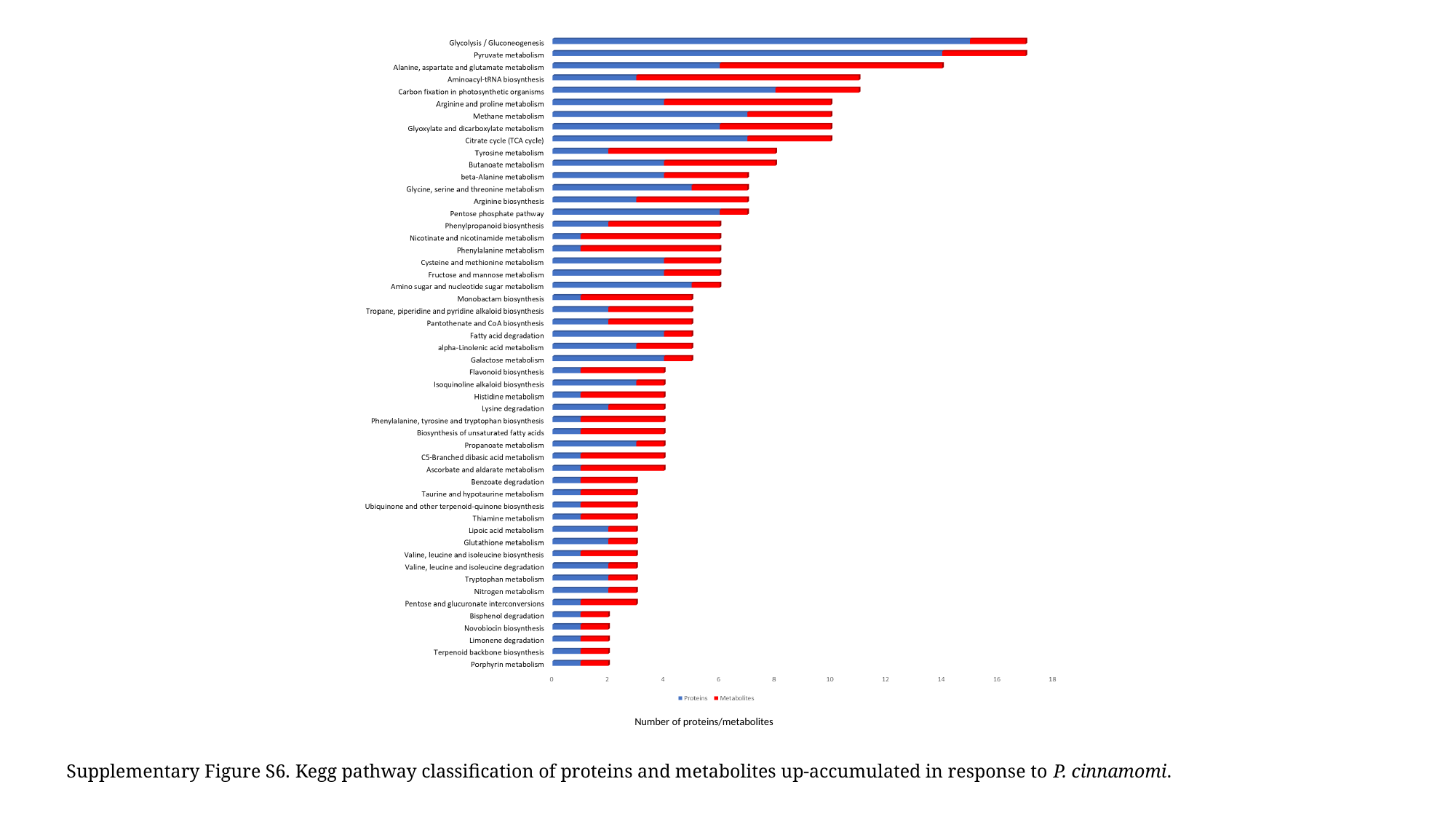

Number of proteins/metabolites
Supplementary Figure S6. Kegg pathway classification of proteins and metabolites up-accumulated in response to P. cinnamomi.

## Slide 7
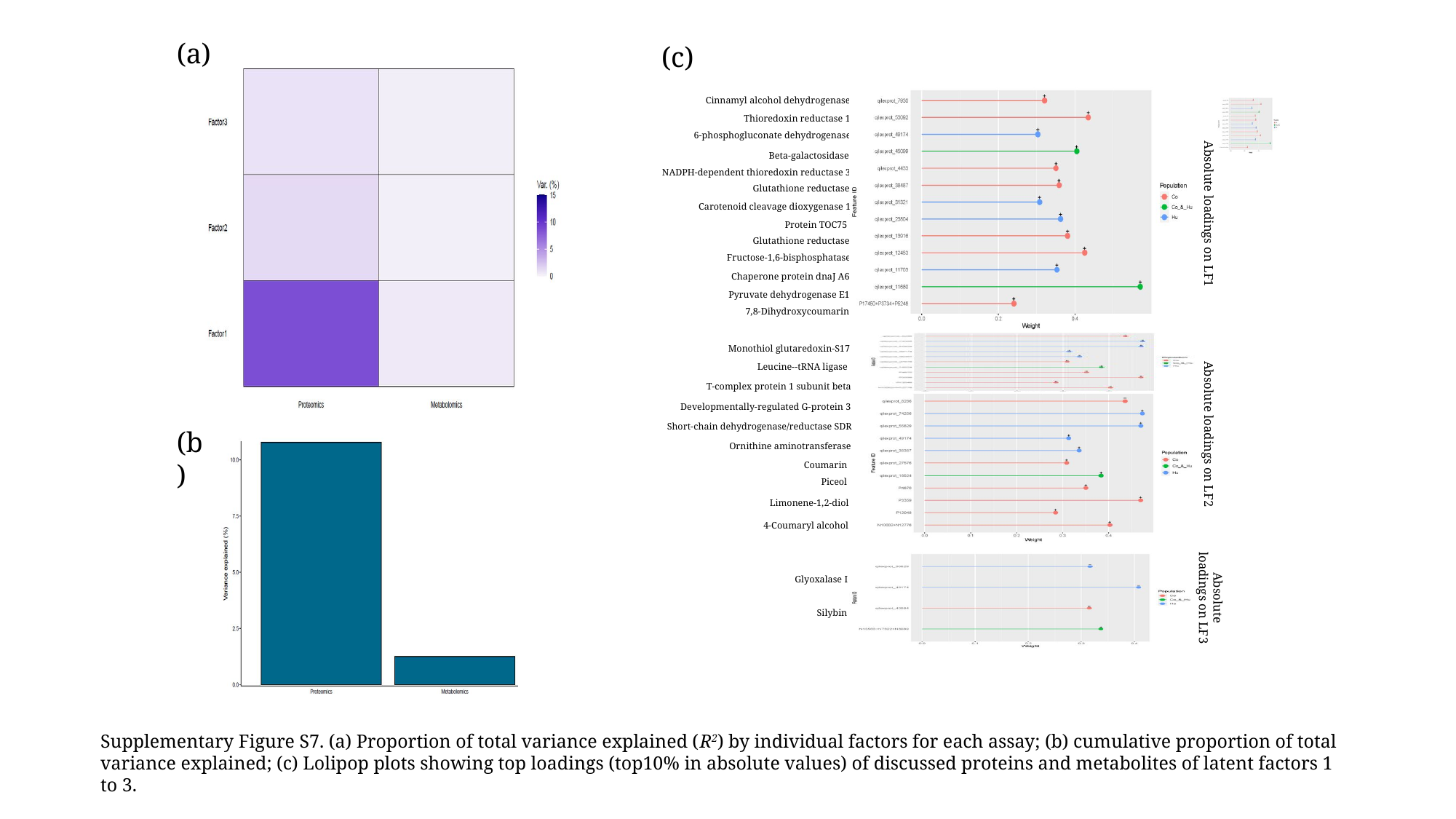

(a)
(c)
Absolute loadings on LF1
Absolute loadings on LF2
Absolute loadings on LF3
Cinnamyl alcohol dehydrogenase
Thioredoxin reductase 1
6-phosphogluconate dehydrogenase
Beta-galactosidase
NADPH-dependent thioredoxin reductase 3
Glutathione reductase
Carotenoid cleavage dioxygenase 1
Protein TOC75
Glutathione reductase
Fructose-1,6-bisphosphatase
Chaperone protein dnaJ A6
Pyruvate dehydrogenase E1
7,8-Dihydroxycoumarin
Monothiol glutaredoxin-S17
Leucine--tRNA ligase
T-complex protein 1 subunit beta
Developmentally-regulated G-protein 3
Short-chain dehydrogenase/reductase SDR
Ornithine aminotransferase
Coumarin
Piceol
Limonene-1,2-diol
4-Coumaryl alcohol
Glyoxalase I
Silybin
(b)
Supplementary Figure S7. (a) Proportion of total variance explained (R2) by individual factors for each assay; (b) cumulative proportion of total variance explained; (c) Lolipop plots showing top loadings (top10% in absolute values) of discussed proteins and metabolites of latent factors 1 to 3.
